# Economic evaluation comparing Endobronchial Ultrasound-guided Transbronchial Needle Aspiration (EBUS-TBNA) and surgical mediastinoscopy for mediastinal staging of lung cancer: a systematic review

**DOI:** 10.1186/s43046-026-00396-1

**Published:** 2026-08-11

**Authors:** Zainib Amirah Anwar, Airy Anak Andrew Atoi, Catherine Soo Shen Chan, Dhinagar Selgal Raddy, Edwin De Cruz, Rafidah Lamit, Nabilah Ayob, Suzana Awg Piut, Mohd Hanafiah Ahmad Hijazi, Abdul Rahman Ramdzan, Larry Ellee Nyanti

**Affiliations:** Faculty of Medicine & Health Sciences, Universiti of Malaysia Sabah, Kota Kinabalu, Malaysia

## Abstract

**Introduction:**

Mediastinal sampling is essential for diagnosing and staging thoracic diseases, particularly non-small cell lung cancer (NSCLC). Traditional mediastinoscopy has been the gold standard; however, Endobronchial Ultrasound-guided Transbronchial Needle Aspiration (EBUS-TBNA) has emerged as a minimally invasive alternative. This systematic review compares the economic evaluations of EBUS-TBNA to determine their cost-effectiveness, healthcare resource use, and overall economic impact.

**Methodology:**

A systematic review was conducted following PRISMA guidelines. Databases searched included PubMed and ScienceDirect, using keywords such as “EBUS-TBNA,” “mediastinoscopy,” and “cost-effectiveness.” Inclusion criteria focused on studies evaluating studies comparing EBUS-TBNA with traditional mediastinoscopy, with direct economic comparisons between EBUS-TBNA and traditional mediastinoscopy. Articles published in English within the last 10–15 years were included, covering randomized controlled trials, cohort studies, and economic modeling analyses. Four studies met the eligibility criteria after screening and full-text review.

**Results:**

The review revealed that EBUS-TBNA consistently demonstrated lower costs and better cost-effectiveness compared to TMC. In the study by Steinfort et al. [1], EBUS-TBNA reduced costs by AU$5,898 per patient compared to mediastinoscopy. Similarly, Chouaid et al. [2] reported cost savings of €1,450 per patient in France. EBUS-TBNA avoided mediastinoscopy in 80% of cases while maintaining high diagnostic accuracy. Sensitivity analyses confirmed that EBUS-TBNA was the most cost-effective when its sensitivity exceeded 17% and mediastinal lymph node metastases (MLNM) prevalence was below 79%.

**Discussion:**

EBUS-TBNA offers significant economic and clinical advantages over traditional mediastinoscopy. It reduces the need for invasive procedures, minimizing patient risk and healthcare costs. Its combination with diagnostic tools like PET-CT further enhances its value, as seen in Søgaard et al. [3], where this approach yielded the most cost-effective outcomes for NSCLC staging. While most studies relied on modeled populations, the findings are robust across diverse healthcare settings. Limitations include the lack of real-world long-term analyses and variability in healthcare systems.

**Conclusion:**

This systematic review highlights that EBUS-TBNA is a cost-effective and clinically valuable alternative to traditional mediastinoscopy for mediastinal sampling. Its integration into clinical practice can improve resource utilization and patient outcomes while significantly reducing healthcare costs. Future research should explore its long-term cost-effectiveness and broader applicability in diverse healthcare settings. EBUS-TBNA represents a critical advancement in the diagnostic staging of thoracic diseases.

## Introduction

Mediastinal lymph node staging is critical in guiding management decisions in lung cancer patients. While surgical mediastinoscopy was traditionally the gold standard for mediastinal lymph node sampling, Endobronchial Ultrasound-guided Transbronchial Needle Aspiration (EBUS-TBNA) has become the guideline-recommended first-line diagnostic tool over the past decade due to its minimally invasive nature and high diagnostic accuracy [4]. While mediastinoscopy is performed through incisions penetrating the chest wall under general anesthesia EBUS-TBNA is minimally invasive, permitting mediastinal nodal staging under real-time endobronchial ultrasound guidance through a flexible bronchoscope [5].

According to studies, EBUS-TBNA offers similar outcomes as surgical mediastinoscopy [6] with less complications [7], mitigates surgery, hospital stays, and complications, lowering costs [8]. In addition, a systematic review on economic evaluation studies conducted by Motta et al. suggested that EBUS-TBNA is cheaper than mediastinoscopy for mediastinal lung cancer staging [9].

The main goal of this systematic review is to compare the economic evaluations of EBUS-TBNA and surgical mediastinoscopy for mediastinal staging. It aims to explore how these techniques differ in terms of cost-effectiveness, healthcare resource use, and overall economic impact. By reviewing the evidence, this study seeks to highlight which method offers better value while maintaining high diagnostic accuracy, helping to guide clinical decisions and healthcare policies.

## Methodology

This systematic review follows the PRISMA (Preferred Reporting Items for Systematic Reviews and Meta-Analyses) guidelines to ensure transparency and rigor. A comprehensive search was conducted using two databases: PubMed and ScienceDirect. The search employed the following keywords and their combinations: “mediastinal sampling,” “EBUS-TBNA,” “Endobronchial Ultrasound-guided Transbronchial Needle Aspiration,” “Traditional Mediastinoscopy,” “cost-effectiveness,” “economic evaluation”. Boolean operators (AND, OR) were utilized to optimize the search string. Additional filters were applied to refine the search.

The inclusion criteria were open-access articles with (1) studies comparing EBUS-TBNA with traditional mediastinoscopy; (2) articles reporting on economic evaluations, including cost-effectiveness, cost-utility, or cost–benefit analyses; (3) studies published in English; and (5) randomized controlled trials, cohort studies, or modeling studies that include cost or resource utilization data.

The exclusion criteria were: (1) articles evaluating only one of the techniques without direct comparison; (2) studies lacking economic data or reporting only clinical outcomes; (3) narrative reviews, editorials, conference abstracts, and case reports; and (4) studies conducted in settings not applicable to current healthcare systems.

The search results were screened in two stages: title and abstract screening followed by full-text review to determine final eligibility. Data extraction focused on study characteristics, intervention details, costs, and outcomes to facilitate a detailed synthesis of the economic evaluation of EBUS-TBNA versus mediastinoscopy.

## Results

**Fig. 1:**
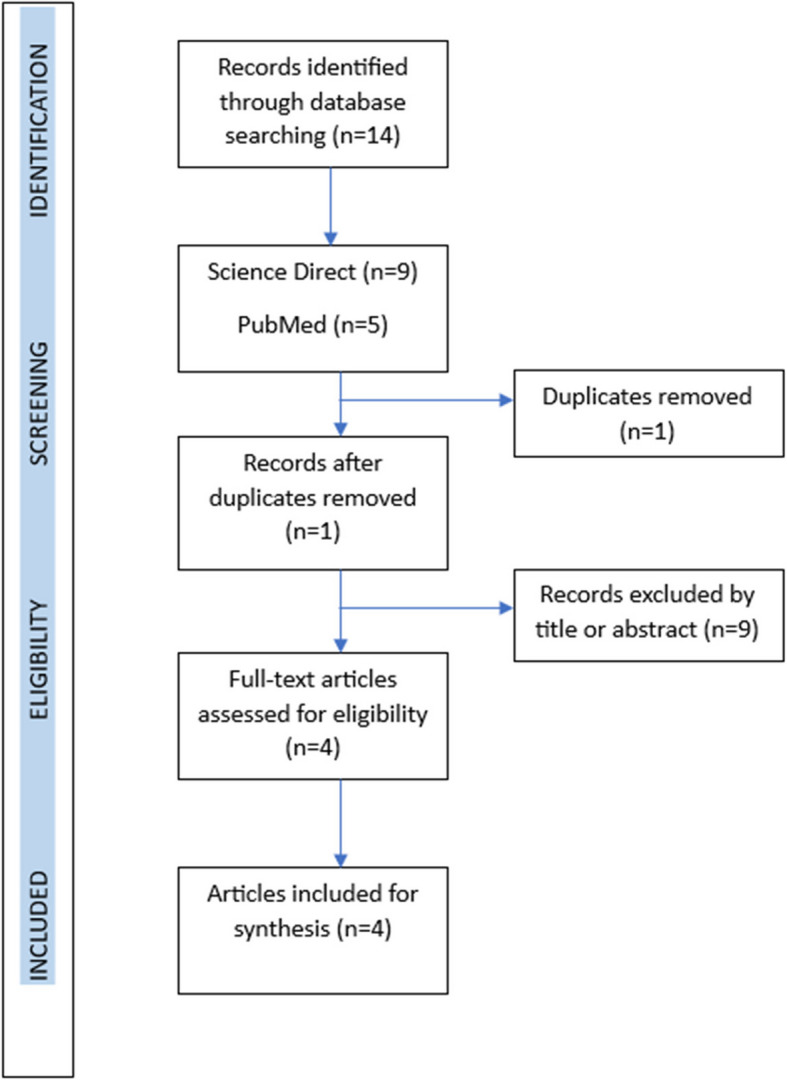
Flow of identification and selection of articles

A total of 14 articles were found during the initial search of ScienceDirect and PubMed databases. Out of them, 1 article was removed due to duplication, subsequently, 9 articles were excluded after the title and abstract screening, and 4 articles underwent full manuscript review and study characteristics as Table 1. Ultimately, 4 articles were accepted in this review (Fig. 1).

**Table 1:** Study characteristics

Author	Country	Type of evaluation	Population (N)	Type of study	Model perspective	Time horizon	Sensitivity analysis
Steinfort et al., 2010 [1]	Australia	Cost–benefit analysis	Hypothetical NSCLC population based on institutional data	Decision-tree analysis	Australian healthcare system	Not explicitly mentioned	Yes, one- and two-way sensitivity analyses
Søgaard et al., 2013[3]	Denmark	Cost-effectiveness	NSCLC patients (model-based)	Decision-analytic	Danish healthcare system	5 years	Yes, deterministic and probabilistic sensitivity analysis
Czarnecka-Kujawa et al., 2016	Canada	Cost-effectiveness	Hypothetical NSCLC patients with clinical N0 disease	Decision-analytic	Canadian healthcare system	Lifetime	Yes, one- and two-way sensitivity analyses
Søgaard et al., 2013 [2]	France	Cost-effectiveness	Patients with NSCLC undergoing mediastinal staging (163) patients	Prospective multicenter trial	French healthcare system	Not explicitly mentioned	Yes, varying costs and procedure types

### Decision tree analysis

In the 2010 study conducted at a major teaching hospital in Melbourne, Australia, the decision-tree analysis method was utilized to explore the cost–benefit of minimally invasive staging for non-small cell lung cancer (NSCLC) [1]. The analysis compares the downstream costs of EBUS-TBNA, conventional TBNA, and surgical mediastinoscopy with the real costs derived from actual patient data. One- and two-way sensitivity analyses were also implemented to account for potential variations in input parameter values.

As a result, initial evaluation with EBUS-TBNA (with negative results being surgically confirmed) was the most cost-beneficial approach (AU$2961) in comparison with EBUS-TBNA (negative results not surgically confirmed) ($3344), conventional TBNA ($3754), and mediastinoscopy ($8859). The base-case analysis revealed that for mediastinal lymph node metastases (MLNM) prevalence under 79%, omitting surgical confirmation of negative EBUS-TBNA results was cost-saving. However, when EBUS-TBNA sensitivity exceeded 93%, confirming negative results was no longer cost-beneficial. The threshold analysis indicated that EBUS-TBNA without confirmation remained least costly if sensitivity was over 17%. Conventional TBNA became more cost-effective than EBUS-TBNA if its sensitivity exceeded 88%. It was also considered cheaper than mediastinoscopy in the absence of EBUS-TBNA as long as the sensitivity remained greater than 15%. Conclusively, EBUS-TBNA’s sensitivity for detecting disease had the largest impact on cost, while the prevalence of MLNM determined whether surgical confirmation of negative EBUS-TBNA results remained cost-beneficial. Additionally, in two-way sensitivity analyses, which varied both the prevalence of MLNM and the sensitivity of EBUS-TBNA, EBUS-TBNA remained the least costly approach.

Another study in 2013 also utilized the decision tree analysis to evaluate the expected costs and outcomes of alternative approaches for lung cancer staging [3]. Six alternatives were defined from relevant combinations of mediastinoscopy, endoscopic or EBUS-TBNA, EUS-FNA (endoscopic ultrasound with fine needle aspiration), and combined PET-CT with F18-fluorodeoxyglucose. Patients without distant metastases and central or contralateral nodal involvement (N2/N3) were considered for thoracotomy. The cost-effectiveness analysis was based on the net present value of expected costs and life years accrued over a period of 5 years.

As a result, in terms of cost, strategy D which consists of sending all patients to PET-CT followed by the confirmation of positive findings on central or contralateral nodal involvement appeared to be the least costly, whereas strategy A with a simpler strategy of mediastinoscopy appeared to be the most expensive. This is vastly due to the price of mediastinoscopy being four times higher than the price of PET-CT, and the number of patients who underwent futile thoracotomy, followed by costly nonsurgical treatment. On the other hand, in terms of outcomes, strategy C which includes sending all patients to EBUS-TBNA appeared to be the best, with an average gain of 1.292 life years, whereas strategy B by sending all patients to EUS-FNA resulted in the least life year gain of 1.275. There was not much difference in the outcome between strategies because it had little effect in terms of survival and there is no single strategy that was able to identify all patients with a true indication for thoracotomy. To choose between the two best strategies in terms of cost and life years gain, strategy D and strategy C, the incremental cost-effectiveness ratio associated with moving from strategy D to strategy C was estimated at 188,461 million euros per life year. In other words, strategy D by sending all patients to PET-CT with confirmation of positive findings on nodal involvement by EBUS appeared to be the most cost-effective method for lung cancer staging.

### Decision-analytic framework

Using a decision-analytic framework, Czarnecka-Kujawa et al. (2016) modeled the health outcomes and costs related with four mediastinal staging options for NSCLC. Using probability to replicate a hypothetical cohort of patients with biopsy-proven or suspected NSCLC with clinical N0 illness, the model synthesized clinical and economic evidence. Among the strategies assessed were no invasive staging, EBUS-TBNA alone, mediastinoscopy alone, and EBUS-TBNA followed by mediastinoscopy should EBUS-TBNA prove negative. Assuming a lifetime horizon, the model included expenses stated in 2015 Canadian dollars and quality-adjusted life years (QALYs) as the evaluation metric. Other presumptions included that all patients were suitable candidates for surgery and that invasive staging methods were absolutely specific. Key factors, such the prevalence of nodal metastases and the sensitivity of EBUS-TBNA, were evaluated using sensitivity studies both one- and two-way. Based on the particular traits of the patient population and healthcare environment, this framework let the authors assess the cost-effectiveness and best staging plan for NSCLC (Czarnecka-Kujawa et al., 2017 [10]).

The study found that EBUS-TBNA with confirmatory mediastinoscopy yielded the highest QALYs at 5.88, with an incremental cost-effectiveness ratio (ICER) of $26,000 per QALY compared to no invasive staging, making it cost-effective based on a CAD $80,000/QALY threshold. Sensitivity analyses showed that EBUS-TBNA alone was cost-effective for moderate probabilities of metastasis (pN2), while the addition of mediastinoscopy was preferred for higher probabilities (pN2 > 57%) (Czarnecka-Kujawa et al., 2016).

### Two-step methodology

Another study in France by Chouaid et al. [2] employed a two-step methodology to evaluate the clinical efficacy and cost-effectiveness of EBUS-TBNA for preoperative staging of NSCLC. The first step involved training and certifying participating centers to perform the EBUS-TBNA procedure, ensuring a high benchmark of technical proficiency. In the second step, a prospective, multicenter trial was conducted with 163 patients who had confirmed or suspected NSCLC requiring mediastinal lymph node staging. All patients underwent EBUS-TBNA as the initial diagnostic procedure, and those with negative or inconclusive results underwent further evaluation with mediastinoscopy or surgery.

The study adopted a cost-effectiveness analysis from the perspective of the French healthcare system, focusing on direct costs related to the diagnostic pathway. Healthcare resource consumption was assessed prospectively, and costs were derived from national healthcare tariffs and diagnosis-related group fees. Sensitivity analyses were conducted to test the robustness of the results, including variations in the frequency of metastatic disease, the use of general anesthesia, and procedural costs. It showed that the cost savings persisted even when EBUS-TBNA was performed under general anesthesia or if procedural costs increased by 30% [2].

The study found that EBUS-TBNA was highly cost-effective, achieving an average cost savings of €1,450 per patient compared to mediastinoscopy. Furthermore, EBUS-TBNA avoided the need for mediastinoscopy in 80% of patients, significantly reducing healthcare resource utilization. Diagnostic accuracy was also high, with a positive predictive value (PPV) of 100% and a negative predictive value (NPV) of 90% [2].

The cost data findings shown in Table 2 from the studies indicate diverse approaches to cost accounting across different healthcare systems and procedures. Steinfort et al. [1], using hospital records from Melbourne, Australia, calculated direct healthcare costs with a focus on procedure costs, sedation, and day-care for minimally invasive techniques. The costs were updated to 2009 values, reporting AU$ 2,961 for EBUS-TBNA and AU$ 8,859 for mediastinoscopy, with a 3% annual inflation rate. Søgaard et al. [3] utilized the Danish Lung Cancer Registry and national tariffs to evaluate diagnostic and survival costs for supplemental staging, estimating a willingness-to-pay threshold of €30,000 per procedure, though specific procedure costs were not detailed.

**Table 2:** Cost data

Author	Type of costs	Cost items	Cost data sources	Year accounted	Currency	Unit	Inflation rate	Discount rate	Willingness-to-pay threshold	EBUS-TBNA ($)	Mediastinoscopy ($)
Steinfort et al., 2010 [1]	Direct healthcare costs	Procedure costs, day-care, sedation	Hospital records (Melbourne)	2007/2008 updated to 2009	AUD ($)	Per procedure	3% annually	Not specified	Not specified	AU$2,961	AU$ 8,859
Søgaard et al., 2013 [3]	Direct and indirect costs	Diagnostic procedures, survival costs	Danish Lung Cancer Registry, National Tariffs	Not specified	Euro (€)	Per procedure	Not specified	Not specified	€30,000	Not specified	Not specified
Czarnecka-Kujawa et al., 2017 [10]	Direct costs, QALY-based costs	Procedures, chemotherapy, radiation, surgery	Hospital records (Toronto), Canadian Cancer Registry	2015	CAD ($)	Per patient/procedure	Adjusted to 2015	Not specified	CAD $ 80,000/ QALY	CAD $13,727	CAD $ 18,143
Søgaard et al., 2013 [2]	Direct medical costs	Procedure costs, hospital costs	National healthcare tariffs	Not specified	Euro (€)	Per patient	Not specified	Not specified	Not specified	€120 (local anesthesia)	€ 2,300

Czarnecka-Kujawa et al. (2016) analyzed direct costs including procedures, chemotherapy, and surgery from Toronto hospital records, adjusted to 2015 Canadian dollars, reporting CAD $13,727 for EBUS-TBNA and CAD $18,143 for mediastinoscopy, with a willingness-to-pay threshold of CAD $80,000/QALY. Lastly, Chouaid et al. [2] evaluated direct medical costs from national healthcare tariffs in France, calculating €120 for EBUS-TBNA under local anesthesia and €2,300 for mediastinoscopy, without adjusting for inflation or specifying a willingness-to-pay threshold. These studies illustrate significant variability in cost structures, unit costs, and economic evaluation methods across different healthcare contexts.

Table 3 showed interventions, comparators and outcomes of the studies. Steinfort et al. [1] compared mediastinal staging methods for NSCLC cost–benefit analysis. EBUS-TBNA with surgical confirmation of negative results was the primary intervention. This was compared with TBNA and mediastinoscopy. EBUS-TBNA was the most cost-effective method, costing AU$2,961 per patient compared to AU$3,754 for traditional TBNA and AU$8,859 for mediastinoscopy. The study also found that EBUS-TBNA diagnostic accuracy sensitivity affected cost-effectiveness the most. These findings showed that minimally invasive staging methods like EBUS-TBNA are more cost-efficient and effective than mediastinoscopy, especially when surgical confirmation is needed for negative results.

**Table 3:** Interventions, comparators and outcomes

Author	Intervention	Comparators	Outcome
Steinfort et al., 2010 [1]	EBUS-TBNA for NSCLC staging	Conventional TBNA and surgical mediastinoscopy	EBUS-TBNA was the most cost-beneficial method, with costs of AU$2,961 compared to AU$3,344 (TBNA) and AU$8,859 (mediastinoscopy)
Søgaard et al., 2013 [3]	PET-CT with EBUS-TBNA confirmation	Mediastinoscopy, EBUS-TBNS, EBUS-FNA, and standalone PET-CT	Combined PET-CT with EBUS-TBNA confirmation was cost-effective; and robust across sensitivity analyses with potential savings for Danish healthcare
Czarnecka-Kujawa et al., 2017 [10]	EBUS-TBNA and EBUS-TBNA followed by mediastinoscopy	No invasive staging, mediastinoscopy, and no EBUS-TBNA confirmation	EBUS-TBNA was cost-effective at CAD $26,000/QALY; EBUS-TBNA followed by mediastinoscopy became cost-effective at higher pN2 prevalence (57%)
Chouaid et al., 2019 [2]	EBUS-TBNA for NSCLC staging	Mediastinoscopy as the primary diagnostic method	EBUS-TBNA was cost-effective, avoided mediastinoscopy in 80% of cases, and resulted in savings of €1,450 per patient

A cost-effectiveness analysis by Søgaard et al. [3] assessed additional staging options for NSCLC. EBUS-TBNA was compared to mediastinoscopy and other staging methods, including PET-CT. The study recommended EBUS-TBNA for supplemental staging since it was cost-effective, especially when paired with PET-CT. Deterministic sensitivity studies showed that the intervention improved surgery candidate identification accuracy and cost-effectiveness. These findings suggested that PET-CT and EBUS-TBNA should be used to stage NSCLC because it improves resource usage and clinical outcomes.

Czarnecka-Kujawa et al. (2016) performed a cost-effectiveness analysis to compare different methods of mediastinal staging in NSCLC. The study focused on EBUS-TBNA, with mediastinoscopy performed if EBUS-TBNA results were negative. This intervention was assessed against no invasive staging, EBUS-TBNA alone, and mediastinoscopy alone. The research indicated that EBUS-TBNA followed by mediastinoscopy was the most effective approach, yielding 5.88 QALYs and an ICER of $26,000 per QALY relative to no invasive staging. The analysis indicated that mediastinoscopy alone was less effective, whereas EBUS-TBNA was cost-effective for cases with a moderate likelihood of mediastinal metastasis (pN2). The study found that EBUS-TBNA, followed by mediastinoscopy, provides an effective and cost-efficient approach, especially for patients with a strong clinical suspicion of nodal involvement.

Chouaid et al. [2] performed a prospective multicenter trial to assess the clinical efficacy and cost-effectiveness of EBUS-TBNA for preoperative staging of NSCLC in France. The intervention EBUS-TBNA was evaluated against mediastinoscopy, the conventional standard for mediastinal staging. The study showed that EBUS-TBNA was cost-effective, saving an average of €1,450 per patient compared to mediastinoscopy. EBUS-TBNA eliminated the necessity for mediastinoscopy in 80% of patients, achieving high diagnostic accuracy with a positive predictive value of 100% and a negative predictive value of 90%. The findings indicate that EBUS-TBNA is a cost-effective and minimally invasive alternative to mediastinoscopy, presenting a beneficial option for NSCLC staging in clinical practice.

In Table 4, all studies demonstrate that EBUS-TBNA is a cost-effective strategy for staging NSCLC, consistently outperforming traditional approaches like mediastinoscopy in cost savings and clinical utility. Steinfort et al. [1] and Chouaid et al. [2] highlight substantial cost savings and recommend EBUS-TBNA as a minimally invasive alternative, especially when combined with confirmatory testing for negative results. Søgaard et al. [3] emphasize the robustness of combining PET-CT with EBUS-TBNA under deterministic sensitivity analysis, advocating for its implementation in Denmark. Czarnecka-Kujawa et al. (2016) find EBUS-TBNA cost-effective in most scenarios, with confirmatory mediastinoscopy proving necessary in high-risk cases. While all studies incorporate thorough sensitivity analyses to validate their conclusions, they reveal critical factors influencing cost-effectiveness, such as disease prevalence and procedural sensitivity. Collectively, the findings support the consideration of EBUS-TBNA for NSCLC staging, with targeted refinements in high-risk patient management.

**Table 4:** Cost-effectiveness, cost-savings, and sensitivity analysis results

Author	Cost-effectiveness and cost-savings results	Sensitivity analysis results	Conclusions
Steinfort et al., 2010 [1]	EBUS-TBNA cost: AU$2,961; Mediastinoscopy cost: AU$8,859. EBUS-TBNA was the most cost-beneficial option for NSCLC staging	The sensitivity of EBUS-TBNA and the prevalence of mediastinal lymph node metastases (MLNM) were key parameters influencing cost-effectiveness	EBUS-TBNA is cost-beneficial compared to conventional TBNA and mediastinoscopy, especially when negative results are surgically confirmed
Søgaard et al., 2013 [3]	PET-CT with EBUS-TBNA confirmation was cost-effective at a threshold of ~ €30,000, and robust to deterministic sensitivity analysis	The expected value of perfect information (EVPI) per patient was estimated at €52; deterministic sensitivity analysis confirmed the robustness of results across a wide parameter range	Combined PET-CT with EBUS-TBNA confirmation is cost-effective and should be made available for supplemental staging of NSCLC in Denmark
Czarnecka-Kujawa et al., 2017 [10]	EBUS-TBNA: CAD $26,000/QALY. EBUS-TBNA followed by mediastinoscopy cost-effective at pN2 prevalence ≥ 57%. EBUS-TBNA without mediastinoscopy dominated mediastinoscopy	Prevalence of pN2 disease and EBUS-TBNA sensitivity significantly affected cost-effectiveness thresholds	EBUS-TBNA is cost-effective for staging patients with early NSCLC. Confirmatory mediastinoscopy should be added in high-risk patients with negative EBUS-TBNA
Chouaid et al., 2019 [2]	EBUS-TBNA saved €1,450 per patient compared to systematic mediastinoscopy. Cost savings persisted even with 30% higher costs or use of general anesthesia for EBUS-TBNA	Sensitivity analysis confirmed cost savings with cost increases or changes in procedural approaches	EBUS-TBNA is cost-effective for NSCLC staging, reducing the need for mediastinoscopy and providing substantial savings for the French healthcare system

### Quality assessment

All four articles were appraised using Consolidated Health Economic Evaluation Reporting Standards 2022 [11] (CHEERS 2022) (Appendix A). The articles demonstrate strong adherence to the CHEERS checklist in most reporting domains, including clear documentation of study parameters, analytic methods, results, and uncertainty through sensitivity analyses. They effectively disclose funding sources and conflicts of interest, ensuring transparency. However, some limitations were identified. None of the studies explicitly report engagement with patients, stakeholders, or communities in their design, which is a missed opportunity to enhance relevance and applicability. Additionally, Steinfort et al. [1] and Chouaid et al. [2] lack explicit reporting of time horizons and discount rates, which could impact the interpretation of long-term cost-effectiveness. None of the studies explicitly report how heterogeneity or distributional effects were addressed, limiting insights into subgroup variability and equity considerations. Overall, while the studies provide robust economic evaluations, addressing these gaps would strengthen their comprehensiveness and applicability in informing healthcare decisions.

## Discussion

The findings of this systematic review underscore the economic and clinical advantages of EBUS-TBNA compared to traditional mediastinoscopy for mediastinal sampling in the staging of NSCLC. Although the economic advantage of EBUS-TBNA has been previously described, this review consolidates evidence across multiple healthcare systems and highlights key determinants influencing cost-effectiveness. Such synthesis is particularly valuable for healthcare decision-makers considering the transition from surgical to minimally invasive staging strategies.

Across multiple studies, EBUS-TBNA consistently demonstrated superior cost-effectiveness and safety while maintaining high diagnostic accuracy. Steinfort et al. [1] highlighted that EBUS-TBNA reduced costs by up to AU$ 5,898 per patient compared to TMC, while Chouaid et al. [2] reported savings of €1,450 per patient in the French healthcare system. Additionally, Søgaard et al. [3] emphasized that a combination of PET-CT and EBUS-TBNA confirmation was the most cost-effective strategy, offering substantial savings for the Danish healthcare system. These cost savings were robust across sensitivity analyses, affirming the reliability of the findings in diverse clinical and economic scenarios.

Clinically, EBUS-TBNA reduced the need for invasive procedures like mediastinoscopy in 80% of cases, minimizing patient risk and improving outcomes. For high-risk patients with MLNM prevalence exceeding 57%, combining EBUS-TBNA with surgical confirmation added significant diagnostic value, as demonstrated by Czarnecka-Kujawa et al. [10]. Importantly, the sensitivity of EBUS-TBNA and the prevalence of MLNM emerged as critical determinants of its cost-effectiveness. Threshold analyses confirmed that EBUS-TBNA was the most cost-beneficial option when its sensitivity exceeded 17% and MLNM prevalence was below 79%, supporting its consideration as a primary diagnostic tool.

The findings suggest important implications for clinical practice. EBUS-TBNA may offer economic and clinical advantages for mediastinal sampling, particularly in appropriately resourced healthcare settings.

The studies, while based in high-income healthcare settings, offer valuable insights for low- and middle-income countries (LMICs). EBUS-TBNA involves significant initial costs for specialized equipment and trained staff, but its minimally invasive approach may lead to lower overall expenses by decreasing hospital stays, reducing the need for surgeries, and lowering complication rates. The cost-effectiveness of EBUS-TBNA in LMICs may largely rely on procedural volume, operator expertise, and the availability of diagnostic support. Therefore, implementation is likely more practical in tertiary referral centers that have established bronchoscopy services compared to decentralized healthcare systems.

Although EBUS-TBNA has emerged as a standard procedure in numerous high-income nations, its implementation ought to be informed by local economic conditions, the skill level of the workforce, and the existing healthcare infrastructure. This review offers valuable insights for policymakers as they assess the financial sustainability of investing in minimally invasive staging technologies within their health systems.

The systematic review's strengths include a comprehensive literature search and robust sensitivity analyses, which enhance the generalizability of its findings. However, most studies relied on modeled or hypothetical populations, which may not fully capture real-world variability. Additionally, the long-term cost implications of integrating EBUS-TBNA with complementary techniques, such as PET-CT, remain underexplored.

Future research should focus on conducting long-term cost-effectiveness analyses of EBUS-TBNA, especially in combination with emerging diagnostic tools like PET-CT. Economic outcomes are closely tied to the quality of procedures. Therefore, performance indicators like diagnostic yield, complication rates, tissue sampling adequacy, and operator proficiency significantly impact clinical effectiveness and cost-efficiency. Future research that incorporates these quality metrics into economic evaluations will offer a more thorough assessment of value in mediastinal staging methods.

Further investigation into its scalability and accessibility in low-resource settings is also crucial. Moreover, incorporating patient-centered outcomes such as quality of life and recovery times could complement economic evaluations and provide a more holistic understanding of its benefits.

Overall, this review reaffirms that EBUS-TBNA is a cost-effective and clinically valuable alternative to mediastinoscopy for mediastinal sampling. Its integration into clinical guidelines has the potential to enhance the efficiency and affordability of lung cancer staging, benefiting patients and healthcare systems alike.

## Conclusion

This systematic review suggests that EBUS-TBNA is a cost-effective and clinically advantageous alternative to traditional mediastinoscopy for mediastinal sampling, particularly in the staging of NSCLC. Across various healthcare systems, EBUS-TBNA consistently demonstrated lower costs, reduced hospital stays, and fewer complications while maintaining high diagnostic accuracy. It effectively minimized the need for invasive procedures, reducing patient risk and healthcare expenditures.

The findings also highlight that EBUS-TBNA's cost-effectiveness is strongly influenced by its sensitivity and the prevalence of MLNM. Combining EBUS-TBNA with other diagnostic tools like PET-CT further enhances its economic and clinical value. However, while the current evidence supports the adoption of EBUS-TBNA as the standard approach for mediastinal sampling, long-term analyses, and real-world studies are needed to explore its integration with newer diagnostic modalities and its scalability in diverse healthcare settings.

In conclusion, EBUS-TBNA offers significant economic and clinical benefits, appearing as a cost-effective choice for mediastinal sampling in lung cancer staging. Its integration into clinical practice and healthcare policies has the potential to improve patient outcomes, optimize resource allocation, and reduce healthcare costs, marking a significant step forward in the management of thoracic diseases.

## Data Availability

The datasets analyzed during the current study are available from the corresponding author on reasonable request. All data generated or analyzed during this systematic review are included in this published article.

## Appendix A

### Consolidated Health Economic Evaluation Reporting Standards 2022 (CHEERS 2022)

**Table:**

	**Article **		Steinfort et al., 2010 [1]	Søgaard et al., 2013 [3]	** Czarnecka-Kujawa et al., 2016**	Chouaid et al., 2019 [2]
NO	**Item**	**Guidance for Reporting**	**Reported in Section**			
1	**Title**	Identify the study as an economic evaluation and specify the interventions being compared	Title page	Title page	Title page	Title page
2	**Abstract**	Provide a structured summary that highlights context, key methods, results, and alternative analyses	Abstract	Abstract	Abstract	Abstract
	**Introduction**					
3	Background and objectives	Give the context for the study, the study question, and its practical relevance for decision making in policy or practice	Introduction	Introduction	Introduction	Introduction
	**Methods**					
4	Health economic analysis plan	Indicate whether a health economic analysis plan was developed and where available	Methods	Methods	Methods	Methods
5	Study population	Describe characteristics of the study population (such as age range, demographics, socioeconomic, or clinical characteristics)	Methods	Methods	Methods	Methods
6	Setting and location	Provide relevant contextual information that may influence findings	Introduction	Introduction	Introduction	Introduction
7	Comparators	Describe the interventions or strategies being compared and why chosen	Methods	Methods	Methods	Methods
8	Perspective	State the perspective(s) adopted by the study and why chosen	Methods	Methods	Methods	Methods
9	Time horizon	State the time horizon for the study and why appropriate	Methods	Methods	Methods	**Not clearly mentioned**
10	Discount rate	Report the discount rate(s) and reason chosen	**Not reported**	Methods	Methods	**Not reported**
11	Selection of outcomes	Describe what outcomes were used as the measure(s) of benefit(s) and harm(s)	Methods	Methods	Methods	Methods
12	Measurement of outcomes	Describe how outcomes used to capture benefit(s) and harm(s) were measured	Methods	Methods	Methods	Methods
13	Valuation of outcomes	Describe the population and methods used to measure and value outcomes	Methods	Methods	Methods	Methods
14	Measurement and valuation of resources and costs	Describe how costs were valued	Methods	Methods	Methods	Methods
15	Currency, price date, and conversion	Report the dates of the estimated resource quantities and unit costs, plus the currency and year of conversion	Methods	Methods	Methods	Methods
16	Rationale and description of model	If modelling is used, describe in detail and why used. Report if the model is publicly available and where it can be accessed	Methods	Methods	Methods	**Not applicable (trial)**
17	Analytics and assumptions	Describe any methods for analysing or statistically transforming data, any extrapolation methods, and approaches for validating any model used	Methods	Methods	Methods	Methods
18	Characterizing heterogeneity	Describe any methods used for estimating how the results of the study vary for sub-groups	**Not clearly mentioned**	**Not clearly mentioned**	**Not clearly mentioned**	**Not clearly mentioned**
19	Characterizing distributional effects	Describe how impacts are distributed across different individuals or adjustments made to reflect priority populations	**Not clearly mentioned**	**Not clearly mentioned**	**Not clearly mentioned**	**Not clearly mentioned**
20	Characterizing uncertainty	Describe methods to characterize any sources of uncertainty in the analysis	Sensitivity analysis	Sensitivity analysis	Sensitivity analysis	Sensitivity analysis
21	Approach to engagement with patients and others affected by the study	Describe any approaches to engage patients or service recipients, the general public, communities, or stakeholders (e.g., clinicians or payers) in the design of the study	**Not reported**	**Not reported**	**Not reported**	**Not reported**
	**Results**					
22	Study parameters	Report all analytic inputs (e.g., values, ranges, references) including uncertainty or distributional assumptions	Methods	Methods	Methods	Methods
23	Summary of main results	Report the mean values for the main categories of costs and outcomes of interest and summarise them in the most appropriate overall measure	Results	Results	Results	Results
24	Effect of uncertainty	Describe how uncertainty about analytic judgments, inputs, or projections affect findings. Report the effect of choice of discount rate and time horizon, if applicable	Sensitivity analysis	Sensitivity analysis	Sensitivity analysis	Sensitivity analysis
25	Effect of engagement with patients and others affected by the study	Report on any difference patient/service recipient, general public, community, or stakeholder involvement made to the approach or findings of the study	**Not reported**	**Not reported**	**Not reported**	**Not reported**
	**Discussion**					
26	Study findings, limitations, generalizability, and current knowledge	Report key findings, limitations, ethical or equity considerations not captured, and how these could impact patients, policy, or practice	Discussion	Discussion	Discussion	Discussion
	**Other Relevant Information**					
27	Source of funding	Describe how the study was funded and any role of the funder in the identification, design, conduct, and reporting of the analysis	Acknowledgements	Acknowledgements	Acknowledgements	Acknowledgements
28	Conflicts of interest	Report authors conflicts of interest according to journal or International Committee of Medical Journal Editors requirements	Disclosure	Disclosure	Disclosure	Disclosure

Adapted from Husereau D, Drummond M, Augustovski F, de Bekker-Grob E, Briggs AH, Carswell C, Caulley L, Chaiyakunapruk N, Greenberg D, Loder E, Mauskopf J, Mullins CD, Petrou S, Pwu RF, Staniszewska S; CHEERS 2022 ISPOR Good Research Practices Task Force. Consolidated Health Economic Evaluation Reporting Standards 2022 (CHEERS 2022) Statement: Updated Reporting Guidance for Health Economic Evaluations. BMJ. 2022;376:e067975.
